# Enhancing Quadruple Health Outcomes After Thoracic Surgery: Feasibility Pilot Randomized Controlled Trial Using Digital Home Monitoring

**DOI:** 10.2196/58998

**Published:** 2025-02-12

**Authors:** Mahesh Nagappa, Yamini Subramani, Homer Yang, Natasha Wood, Jill Querney, Lee-Anne Fochesato, Derek Nguyen, Nida Fatima, Janet Martin, Ava John-Baptiste, Rahul Nayak, Mehdi Qiabi, Richard Inculet, Dalilah Fortin, Richard Malthaner

**Affiliations:** 1 Department of Anesthesia and Perioperative Medicine London Health Sciences Centre and St. Joseph Health Care, Lawson Health Research Institute, Schulich School of Medicine and Dentistry Western University London, ON Canada; 2 Department of Nursing, Thoracic Surgery London Health Sciences Centre, Lawson Health Research Institute, Schulich School of Medicine and Dentistry Western University London, ON Canada; 3 Department of Surgery, Division of Thoracic Surgery London Health Sciences Centre and St. Joseph Health Care, Lawson Health Research Institute, Schulich School of Medicine and Dentistry Western University London, ON Canada

**Keywords:** remote monitor, digital home monitoring, continuity of care, quadruple health outcomes, patient satisfaction, caregivers satisfaction, healthcare provider satisfaction, feasibility, RCT, thoracic surgery, postoperative monitoring, surgical recovery, perioperative medicine, patient care, questionnaire

## Abstract

**Background:**

Surgical recovery after hospital discharge often presents challenges for patients and caregivers. Postoperative complications and poorly managed pain at home can lead to unexpected visits to the emergency department (ED) and readmission to the hospital. Digital home monitoring (DHM) may improve postoperative care compared to standard methods.

**Objective:**

We conducted a feasibility study for a randomized controlled trial (RCT) to assess DHM's effectiveness following thoracic surgical procedures compared to standard care.

**Methods:**

We conducted a 2-arm parallel-group pilot RCT at a single tertiary care center. Adult patients undergoing thoracic surgical procedures were randomized 1:1 into 2 groups: the DHM group and the standard of care (control group). We adhered to the intention-to-treat analysis principle. The primary outcome was predetermined RCT feasibility criteria. The trial would be feasible if more than 75% of trial recruitment, protocol adherence, and data collection were achieved. Secondary outcomes included 30-day ED visit rates, 30-day readmission rates, postoperative complications, length of stay, postdischarge 30-day opioid consumption, 30-day quality of recovery, patient-program satisfaction, caregiver satisfaction, health care provider satisfaction, and cost per case.

**Results:**

All RCT feasibility criteria were met. The trial recruitment rate was 87.9% (95% CI 79.4%-93.8%). Protocol adherence and outcome data collection rates were 96.3% (95% CI 89.4%-99.2%) and 98.7% (95% CI 92.9%-99.9%), respectively. In total, 80 patients were randomized, with 40 (50%) in the DHM group and 40 (50%) in the control group. Baseline patient and clinical characteristics were comparable between the 2 groups. The DHM group had fewer unplanned ED visits (2.7% vs 20.5%; *P*=.02), fewer unplanned admission rates (0% vs 7.6%; *P*=.24), lower rates of postoperative complications (20% vs 47.5%, *P*=.01) shorter hospital stays (4.0 vs 6.9 days; *P*=.05), but more opioid consumption (111.6, SD 110.9) vs 74.3, SD 71.9 mg morphine equivalents; *P*=.08) compared to the control group. DHM also resulted in shorter ED visit times (130, SD 0 vs 1048, SD 1093 minutes; *P*=.48) and lower cost per case (CAD $12,145 [US $ 8436.34], SD CAD $8779 [US $ 6098.20] vs CAD $17,247 [US $11,980.37], SD CAD $15,313 [US $10,636.95]; *P*=.07). The quality of recovery scores was clinically significantly better than the controls (185.4, SD 2.6 vs 178.3, SD 3.3; *P*<.001). All 37 patients who completed the intervention answered the program satisfaction survey questionnaires (100%; 95% CI 90.5%-100%). Only 36 out of 80 caregivers responded to the caregiver satisfaction questionnaires at the end of the fourth week post hospital discharge (47.7%; 95% CI 35.7%-59.1%). Health care providers reported a 100% satisfaction rate.

**Conclusions:**

This pilot RCT demonstrates the feasibility of conducting a full-scale trial to assess DHM's efficacy in improving postoperative care following thoracic surgery. DHM shows promise for enhancing continuity of care and warrants further investigation.

**Trial Registration:**

ClinicalTrials.gov NCT04340960; https://clinicaltrials.gov/study/NCT04340960

## Introduction

Recovery following surgical discharge poses significant challenges for patients and their caregivers. This challenge is compounded by the growing practice of discharging patients earlier after surgical procedures, intensifying the postoperative care demands. Moreover, the health care system often operates within a framework of fragmented and poorly integrated services, exacerbating the difficulties faced by patients transitioning from hospital to home after surgery, which can lead to complications and inadequately managed pain, resulting in returns to the hospital or visits to the emergency department (ED) [1-5].

Numerous studies underscore the critical role of postdischarge continuity of care in reducing ED visits and readmission rates (RRs) [6-11]. For instance, Shargall et al [12] successfully implemented an “Integrated Comprehensive Care” program involving allied health care professionals, significantly reducing 30-day RRs among thoracic surgery patients. Similar reductions in RRs have been attributed to patient education, well-coordinated discharge planning, physician follow-up, and in-home visits [13]. Data from Canada highlight that within the first 7 days following surgical discharge, 28.3% of ED diagnoses fell under the Canadian Emergency Department Triage and Acuity Scale (CTAS) IV or V, indicating less urgent or nonurgent cases [14]. It is reasonable to assume that many of these patients could have avoided ED visits by providing appropriate transitional care [15].

To address the needs of patients at a higher risk of postdischarge complications, the concept of continuity of care through digital home monitoring (DHM) emerges as a promising avenue to enhance education, modify behavior, and ultimately achieve improved patient outcomes [9]. With this approach, care teams gain insights into each patient's condition daily or weekly, eliminating the reliance on sporadic office visits, typically occurring only once or twice a year [16]. This continuous and comprehensive view of patient health empowers care teams to make timely adjustments to care plans and proactively engage patients in self-managing care [17]. A virtual care option that extends postdischarge continuity of care offers a viable solution [18-21].

Given the intricacies of providing continuity of care through DHM and the challenges associated with conducting a well-designed randomized controlled trial (RCT) in this context, a pilot study emerges as an essential preliminary step. The primary aim of this pilot study is to assess acceptability, identify logistical requirements, optimize the study design and data collection process, and evaluate readiness for a full-scale trial [22]. Undertaking an RCT that involves continuity of care with a DHM solution is resource-intensive. It raises practical concerns for all stakeholders, including hospital administrators, nurses, clinicians, and patients. Although the primary objective of this pilot study is to examine the feasibility of conducting a comprehensive RCT, this research specifically aims to investigate the feasibility of continuity of care using DHM on postoperative outcomes in patients following thoracic surgery. We hypothesize that continuity of care facilitated by DHM will reduce 30-day ED visits compared to standard care practices.

## Methods

### Overview

A parallel-group, 2-arm pragmatic pilot feasibility RCT was conducted from September 2022 to January 2023 at the London Health Sciences Centre. Participants were allocated 1:1 to receive continuity of care with DHM or standard of care (control) following the discharge after their thoracic surgical procedures. All participants provided written or electronic informed consent using the REDCap (Research Electronic Data Capture) tool hosted at the London Health Sciences Centre (REDCap e-consent). The analyses and reporting adhered to the CONSORT (Consolidated Guidelines of Reporting Trials) guidelines for pilot trials and the CONSORT-EHEALTH (Consolidated Standards of Reporting Trials of Electronic and Mobile Health Applications and Online Telehealth) checklist [23,24].

To execute the components of the DHM interventions, the health care team was trained from May 2021 to August 2022 using the Plan-Do-Study-Act cycle. Inclusion criteria were patients aged 18 years or older, undergoing a thoracic surgical procedure (eg, elective anatomic lung resection or any major foregut procedure, such as an esophagectomy), and the surgeon in agreement with patient enrollment. Accredited thoracic surgeons performed all surgeries. Exclusion criteria were patients with unstable disease processes in the postoperative period (eg, postoperative intensive care unit stay) or those with factors that could impact outcome assessment (eg, cognitive impairment, inability to understand English, and limited access to a telephone, computer, or internet services). Patients were also excluded postoperatively if they had intraoperative or immediate postoperative complications requiring an intensive care unit stay.

Upon enrollment, eligible participants were randomized using the simple randomization feature of REDCap. No stratification factors or blocking were applied. The assignment of groups was concealed until the moment of randomization, at which point REDCap automatically allocated participants to the study arms [25]. All consecutive postoperative patients were approached to participate in the study. The randomization occurred on the day of discharge so that in-hospital care was not biased. Due to the pragmatic nature of the trial, patients, surgeons, clinical navigators (CNs), and other health care providers were not masked in the group allocation.

Preoperative, intraoperative, and postoperative patient management followed standard practices and were similar in both groups. A standardized care pathway for postthoracic surgical procedures was implemented for postoperative pain control involving acetaminophen, nonsteroidal anti-inflammatory drugs, hydromorphone, and adjunct medications, such as pregabalin. These were also prescribed on discharge unless otherwise contraindicated. Patients were monitored continuously after surgery while still in the postanesthesia care unit. While on the surgical ward, routine nursing assessments were conducted per the thoracic unit’s standard of care. Patients in the control group were discharged home without any monitoring, per the current standard of care. Patients who experienced postdischarge complications were instructed to contact their surgeon’s office or visit the hospital ED.

Patients in the DHM group received the same in-hospital care as the control group. In addition, DHM patients signed up for the cloud-based technology platform Vivify Health (Plano, Texas) digital portal with a unique username and password. Through the digital portal, the patient would connect with the CN, who guided the patient through every recovery step. The CN connected, engaged, and educated the patients regarding the recovery pathway. The CN also established clear expectations for patients. Before patients were ready for discharge, patients in the DHM group were given a DHM kit and shown how to use it to maintain continuity of care through the digital care platform. The DHM kit contained a noninvasive blood pressure (NIBP), hemoglobin oxygen saturation (SpO_2_), and heart rate (HR) monitor. The data was transferred to a secured digital care platform through the app. DHM patients had access to speak to one of the health care providers at any time of day (CN or virtual care physician). The CN monitored the dashboard from 8 AM to 4 PM After 4 PM, the CN handed over the monitoring dashboard to one of the preassigned physicians (ie, virtual care physicians). Both the CN and physicians were trained in the platform. The health care provider used the digital platform to communicate, engage, and manage patients remotely and efficiently.

Patients measured their vital signs for 2 weeks. The patient also had daily scheduled video calls on days 1-15 after hospital discharge and on an as-needed basis from days 16-30. During the video calls, patients interacted with the CN and responded to symptom questionnaires. The CN organized unscheduled video visits on days without planned virtual visits if they detected changes in patient vital signs or recovery symptom questionnaires requiring follow-up. During virtual visits, the CN discussed any symptoms the patient was experiencing, evaluated their wounds, and obtained a picture if needed. The CN monitored the digital care platform dashboard from the provider side, with an alert for NIBP, HR, SpO_2_, wound concerns, home medications, and pain. Alerts were displayed in a color-coded fashion on the dashboard. The CN also monitored the patient’s symptoms and identified any changes from the patient’s baseline. The CN called a preassigned clinician (ie, the patient’s surgeon, a study physician, or a nurse practitioner) if any of the patient’s symptoms required medical attention. Physicians could add or modify treatments as needed, and if required, they could have the patient come to an outpatient or ED facility for evaluation or management. Instructions were provided for the patient to call an emergency number (ie, 911) in collaboration and consultation with a physician if appropriate if any symptom indicated immediate distress. The CN and patients were just one button or “mouse click” away from each other, with multiple options to communicate by phone, SMS text message, email, or the virtual care platform (video chat). All these modes of communication were through a secured platform. The CN monitored and intervened by providing patients with advice and next steps if they had health concerns. Self-help educational videos were also available for patients.

This RCT was conducted as a pilot study, with a primary emphasis on assessing feasibility outcomes, which include trial recruitment, protocol adherence, and data collection. We followed the traffic light approach criteria for reporting feasibility outcomes [25-27]. This approach defined (1) feasible (green, 75%-100%) where all feasibility outcomes were met and no protocol modifications were needed; (2) feasible with modification (amber, 50%-75%) where all feasibility outcomes were met or could be met with protocol modifications; and (3) not feasible (red, <50%) where even with protocol modifications, feasibility outcomes could not be met. The clinical outcomes were assessed secondarily to inform the measurement strategy and sample size requirements for a future RCT (ie, by estimating variability, SDs, and prevalence of critical clinical outcomes). Our quadruple health outcome measurement strategy included (1) postoperative outcomes like 30-day ED visits, 30-day RRs, postoperative complications, in-hospital length of stay, 30-day quality of recovery (QoR-40) [28], and postdischarge 30-day opioid consumption; (2) patient-program satisfaction and caregiver satisfaction [29]; (3) health care provider satisfaction; and (4) financial sustainability like cost per case analysis.

Patient-reported outcomes were collected up to 30 days post hospital discharge. Daily data was collected using automatic electronic questionnaires completed digitally and transmitted directly to the REDCap database. Patients also had the option to complete daily questionnaires by video or telephone with a CN. The questionnaires were completed on a smartphone, tablet, or personal computer. Masked assessors verified the data in the REDCap database. Information regarding the 30-day ED visits, 30-day RRs, postoperative complications, postdischarge 30-day opioid consumption, and in-hospital length of stay was obtained from electronic medical records. The patient-program satisfaction survey consisted of 9 questions collected by the research assistant at the end of the 30 days in the DHM group. Patient agreement or satisfaction with statements was recorded on a 5-point scale (from 1=strongly disagree to 5=strongly agree) using a checkmark (✓), with a higher score indicating a higher level of patient agreement or satisfaction. The caregiver survey consists of 17 “Yes” or “No” questions collected by the research assistant at the end of the 30 days in the RPM program in both the DHM and control groups. The satisfaction survey for health care providers comprised 9 questionnaires, addressed at the project's conclusion through the Microsoft Teams survey link and disseminated via electronic mail. Case costing data consisting of the average direct surgical and nonsurgical inpatient costs was obtained for the DHM group and control groups according to the Ontario case costing initiative methodology for 2019-2020 data [30].

The following factors were considered in creating the 5 grades of interventions during the postoperative follow-up using RPM programs: phone calls, video calls, asynchronous messages, self-help educational materials, the amount of time the CN spent addressing the patient’s concerns, and escalation to the virtual care physician. The definition of levels of digital health intervention: (1) no intervention and no assessment; (2) no intervention, but the automatic collection of signs, symptoms, and vital signs questionnaires; (3) mild intervention, wherein the CN spends less than 15 minutes with the patient; (4) moderate intervention, characterized by the CN spending 15-30 minutes with the patient; and (5) severe intervention, involving either the CN spending more than 30 minutes with the patient or the situation being escalated to a virtual care physician for further management.

Based on previous data, at least 70 measured participants were required to estimate SD with enough precision for future sample size calculations [31]. We aimed to recruit and obtain outcome data from 80 patients (40 per group), allowing for an attrition rate of approximately 15%. This sample size was also consistent with recommendations regarding the minimum number of participants required to identify feasibility issues [32]. We used “intention-to-treat” analysis. No formal comparison between the study arms was undertaken for outcomes, given that this is a feasibility study. Quantitative secondary outcome measures were summarized descriptively using appropriate summary statistics in the result section and by the trial arm in the tabular column. Continuous variables were reported as mean, standard deviation, and median (range), as appropriate. Categorical variables were reported as counts and percentages. Statistical analyses were performed using GraphPad Prism (GraphPad) software.

### Ethical Considerations

This study was formally registered with ClinicalTrials.gov (NCT04340960) and received full board review and approval from the institutional research ethics board at Western University (HSREB 114886). All individual participants involved in the study provided informed consent. Furthermore, appropriate measures were implemented to maintain the confidentiality and anonymity of patient data throughout the research. The study posed no significant risks to the participants, who kept the right to withdraw without facing repercussions regarding their standard of care. Ultimately, no financial compensation was offered to the participants involved in the study.

## Results

**Figure 1:**
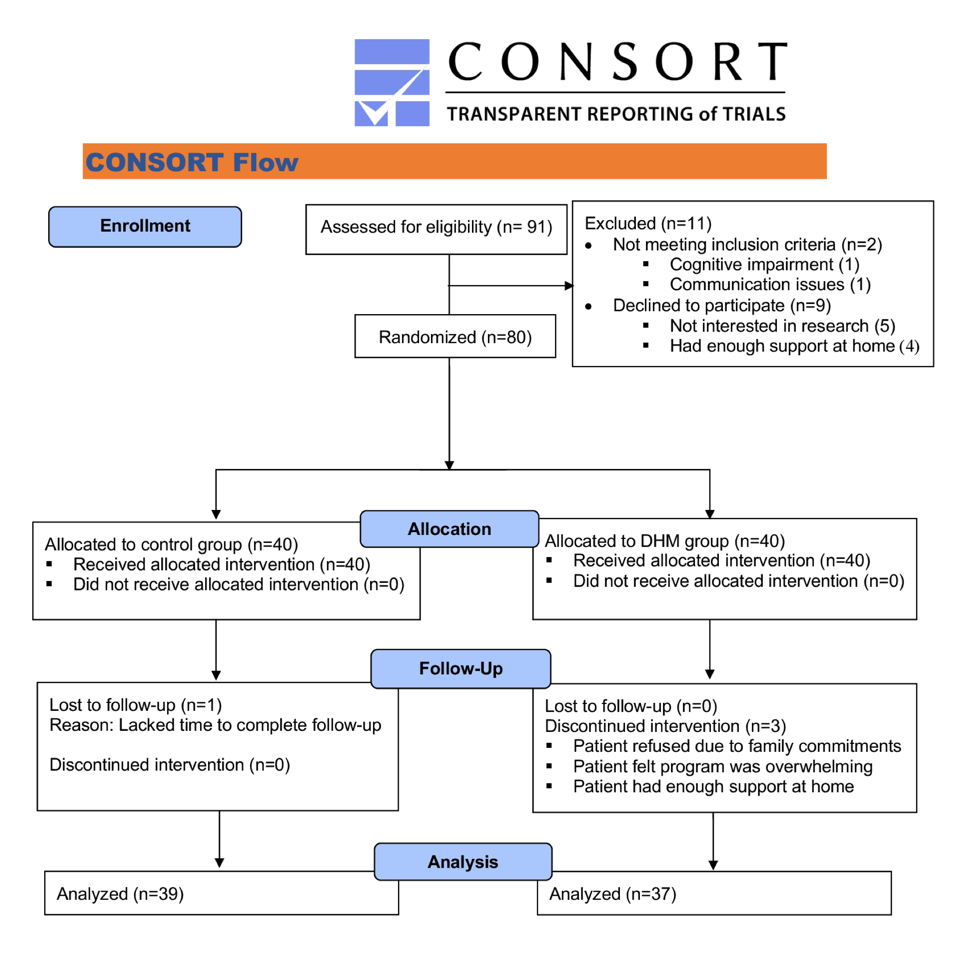
CONSORT (Consolidated Standards of Reporting Trials) flow diagram. DHM: digital home monitoring.

A total of 91 consecutive patients were considered for inclusion in our study. In total, 80 patients met the inclusion criteria, consented to participate, and were randomized to either the control (n=40) group or the DHM (n=40) group (Figure 1). The 2 groups’ patient demographics and clinical characteristics were similar (Table 1).

**Table 1:** Patient demographic and clinical characteristics.

			Control group		DHM^a^ group			Total	*P* value	
**Age (years), mean (SD)**			65.5 (14.7)		63.3 (15.0)			64.4 (14.8)	.51	
**Gender, n (%)**										.36
		Female		25 (62.5)			20 (50)	45 (56.2)		
		Male		15 (37.5)			20 (50)	35 (43.7)		
**BMI (kg/m^2^), mean (SD)**				30.1 (12.3)			27.8 (5.0)	28.9 (9.40)	.28	
**Outside the London area, n (%)**										.48
		Yes		23 (57.5)			27 (67.5)	50 (62.5)		
		No		17 (42.5)			13 (32.5)	30 (37.5)		
**Disease type, n (%)**										.99
		Primary lung cancer		17 (42.5)	17 (42.5)			34 (42.5)		
		Secondary lung cancer		7 (17.5)	7 (17.5)			14 (17.5)		
		Others		16 (40)	16 (40)			32 (40)		
**PFT, mean (SD) (n)**										
		FEV1^b^		85.8 (18.2) (16)			88.5 (21.1) (19)	87.3 (19.6) (35)	.69	
		DLCO^c^		74.6 (19.6) (16)			77.6 (17.1) (18)	76.2 (18.1) (34)	.64	
**Cancer type, n (%)**										.40
		Malignant		24 (60)	23 (57.5)			47 (58.7)		
		Benign		2 (5)	0 (0)			2 (2.5)		
		Others		14 (35)	17 (42.5)			31 (38.7)		
**Side of surgery, n (%)**										.06
		Right		17 (42.5)			8 (20)	25 (31.2)		
		Left		10 (25)			18 (45)	28 (35)		
		N/A^d^		13 (32.5)			14 (35)	27 (33.7)		
**Type of resection, n (%)**										.30
		Wedge		13 (32.5)	11 (27.5)			24 (30)		
		Segmentectomy		4 (10)	1 (2.5)			5 (6.2)		
		Lobectomy		8 (20)	12 (30)			20 (25)		
		Pneumonectomy		0 (0)	0 (0)			0		
		Pleural		3 (7.5)	0 (0)			3 (3.7)		
		Mediastinal		1 (2.5)	2 (5)			3 (3.7)		
		Foregut procedure		11 (27.5)	14 (35)			25 (31.2)		
**Surgical approach, n (%)**										.17
		Thoracotomy		12 (30)	7 (17.5)			19 (23.7)		
		Laparotomy		8 (20)	5 (12.5)			13 (16.2)		
		VATS^e^		18 (45)	21 (52.5)			39 (48.7)		
		Laparoscopic		2 (5)	7 (17.5)			9 (11.2)		
**Staging (pTNM^f^), n (%)**										.66
		IA/IB		14 (35)			9 (22.5)	23 (28.7)		
		IIA/IIB		4 (10)			8 (20)	12 (15)		
		IIIA/IIIB		3 (7.5)			2 (5)	5 (6.2)		
		IV		2 (5)			1 (2.5)	3 (3.7)		
		Metastatic disease		1 (2.5)			2 (5)	3 (3.7)		
		N/A		16 (40)			18 (45)	34 (42.5)		
**Histology, n (%)**										.95
		Adenocarcinoma		13 (32.5)			12 (30)	25 (31.2)		
		Small cell carcinoma		2 (5)			1 (2.5)	3 (3.7)		
		Metastasis		2 (5)			2 (5)	4 (5)		
		Others		1 (2.5)			0 (0)	1 (1.2)		
		Carcinoid		0 (0)			1 (2.5)	1 (1.2)		
		N/A		22 (55)			24 (60)	46 (57.5)		
**Smoking history, n (%)**										.76
		Quit smoking		21 (52.5)		21 (52.5)		42 (52.5)		
		Active smokers		5 (12.5)		3 (7.5)		8 (10)		
	Nonsmokers			14 (35)	16 (40)			30 (37.5)		

^a^DHM: digital home monitoring.

^b^FEV1: forced expiratory volume at the end of 1 second.

^c^DLCO: diffusing capacity of lung for carbon monoxide.

^d^N/A: not applicable.

^e^VATS: video-assisted thoracoscopy.

^f^pTNM: tumor (T), lymph nodes (N), metastasis (M).

Among the eligible patients who declined enrollment, the most common reason was not being interested in participating in research while receiving care (5.4%), followed by patients having enough support at home for recovery after hospital discharge (4.3%). In total, 3 patients from the DHM group withdrew in the second week after hospital discharge. The first patient withdrew due to family commitments, the second patient felt the program was overwhelming, and the last patient had enough support at home during recovery and decided to withdraw from the program. Only one patient from the control group was lost at the end of the 30-day follow-up period. In total, 76 patients—39 in the control group and 37 in the DHM group—completed the study. Out of 80 caregivers who provided consent for enrollment, only 36 caregivers (16 in the control group and 20 in the DHM group) responded to the caregiver satisfaction questionnaires at the end of the fourth week (47.7%; 95% CI 35.7%-59.1%).

Our study met all green feasibility criteria (Table 2). All 5 thoracic surgeons agreed to have their patients consecutively recruited and adhere to the study protocol. The recruitment rate was 87.9% (95% CI 79.4%-93.8%), and protocol adherence was 96.3% (95% CI 89.4%-99.2%). Data were collected for outcomes in 98.7% (95% CI 92.9%-99.9%) of participants.

**Table 2:** Feasibility outcomes.

	Not feasible (red)^a^	Feasible with modification (amber)^b^	Feasible (green)^c^	Study results
Trial recruitment	<50%	50%-74%	75%-100%	87.91%
Protocol adherence	<50%	50%-74%	75%-100%	96.25%
Outcome data collection	<50%	50%-74%	75%-100%	98.70%

^a^Not feasible (red) <50%: even with protocol modifications, some feasibility outcomes cannot be met.

^b^Feasible with modification (amber) 50%-75%: all feasibility outcomes are met or can be met with protocol modifications.

^c^Feasible (green) 75%-100%: all feasibility outcomes are met; no protocol modifications are needed.

The mean age of the sample was 64.4 (SD 14.8) years, with 56.2% being female, and the mean BMI was 28.9 (SD 9.4) kg/m^2^. Most patients had malignant cancer (58.7%) and primary lung cancer (42.5%). Patients most commonly underwent wedge resection (30%), lobectomy (25%), or foregut procedures (31.2%). The most common surgical approach was video-assisted thoracoscopy (48.7%), followed by thoracotomy (23.7%), and then laparotomy (16.2%).

The mean total length of stay in the hospital was 5.4 (SD 6.6) days (control vs DHM: 6.9, SD 8.8 vs 4.0, SD 2.7), and the incidence of postoperative complications was 33.7% (control vs DHM: 47.5% vs 20%). The total number of ED visits in this sample was 11.2% (control vs DHM: 20.5% vs 2.7%). All these ED visits were unplanned, and the mean time spent in the ED was 894 (SD 1047) minutes (control vs DHM: 1048, SD 1093 vs 130, SD 0). One patient from the DHM group presented to the ED with testicular pain. Patients from the control group presented with abdominal bloating or distension, wound concerns, dysphagia, or pain crises. The total hospital RR for the sample was 6.5% (control vs DHM: 7.6% vs 5.4%). The unplanned hospital RR was 3.9% (control vs DHM: 7.6% vs 0%), and the planned hospital RR was 2.6% (control vs DHM: 0% vs 5.4%), respectively. The mean 30-day morphine equivalent dose opioid consumption was 92 (SD 94.2) mg (control vs DHM: 74.3, SD 71.9 vs 111.6, SD 110.9), and the mean in-hospital cost per case was CAD $14,729 (US $10,227.96; SD CAD $12,702 [US $8820.40]; control vs DHM: CAD $17,247 [US $11,976.49], SD CAD $15,313 [US $10,633.50] vs CAD $12,145 [US $8433.61], SD CAD $8779 [US $6096.23]; Table 3).

**Table 3:** Postoperative outcomes.

	Control group	DHM^a^ group	Total	*P* value
LOS^b^ (days), mean (SD)	6.9 (8.8)	4.0 (2.7)	5.4 (6.6)	.05
Postoperative complications, n (%)	19 (47.5)	8 (20)	27 (33.75)	.01
Unplanned ED^c^ visits, n (%)	8 (20.5)	1 (2.7)	9 (11.25)	.02
Planned ED visits, n (%)	0	0	0	—^d^
Time spent in ED (min), mean (SD)	1048 (1093)	130 (0)	894 (1047)	.48
Unplanned RR^e^, n (%)	3 (7.6)	0	3 (3.9)	.24
Planned RR, n (%)	0	2 (5.4)	2 (2.6)	.23
Total RR, n (%)	3 (7.6)	2 (5.4)	5 (6.5)	.99
30-Day morphine equivalent dose consumption (mg), mean (SD)	74.3 (71.9)	111.6 (110.9)	92.4 (94.2)	.08
Cost per case (CAD $; CAD $1=US $0.69), mean (SD)	17,247 (15,313)	12,145 (8779)	14,729 (12,702)	.07

^a^DHM: digital home monitoring.

^b^LOS: length of hospital stay.

^c^ED: emergency department.

^d^Not applicable.

^e^RR: readmission rate.

**Figure 2:**
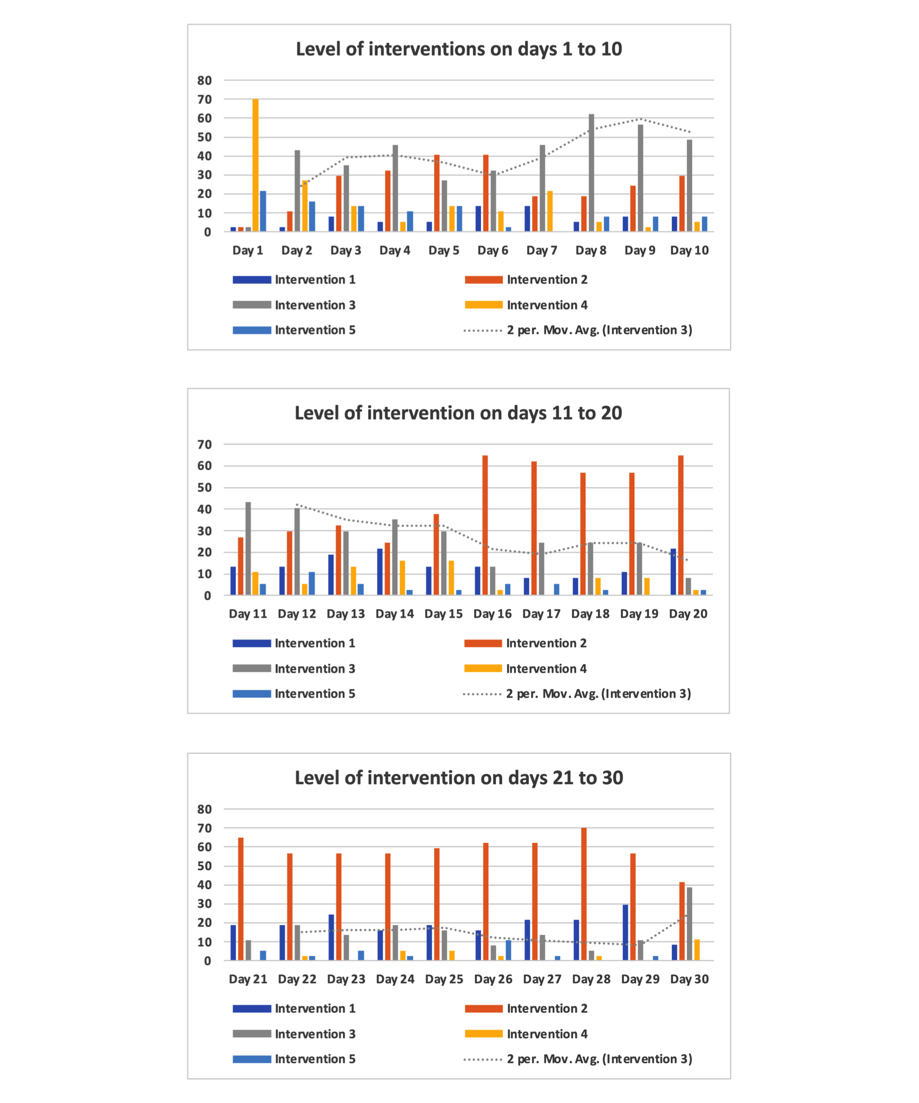
Levels of digital health intervention.

The remote monitoring team most often used level 2 or 3 interventions, except for postdischarge day 1, where intervention level 4 was the most common (Figure 2). Comparing interventions over 0-15 days and 16-30 days revealed that level 2 interventions rose significantly from 26.6% to 59.5% (*P*<.001). In contrast, level 3, 4, and 5 interventions dropped substantially from 38.5% to 16.6%, 15.8% to 3.4%, and 8.6% to 3.2%, respectively (*P*<.001). The most common issues addressed through the digital platform included pain (23%), surgical wound concerns (11%), shortness of breath (10%), diarrhea (7%), medication management (7%), nausea or vomiting (5%), and dizziness (5%; Multimedia Appendix 1).

At 30 days postoperatively, the mean global QoR-40 score for the sample was 181.9 (SD 5.0). The scores for individual domains included emotional status (39.3, SD 1.4), physical comfort (53.5, SD 0.6), psychological support (33.1, SD 1.7), physical independence (22.7, SD 0.6), and pain (33.1, SD 0.4; Table 4).

**Table 4:** Quality of recovery.

	Control group (n=39)	DHM^a^ group (n=37)	Total (N=76)	*P* value
Global QoR-40^b^, mean (SD)	178.3 (3.3)	185.4 (2.6)	181.9 (5.0)	<.001
Emotional status, mean (SD)	38.3 (0.8)	40.4 (0.6)	39.3 (1.4)	<.001
Physical comfort, mean (SD)	53.0 (0.7)	53.9 (0.7)	53.5 (0.6)	<.001
Psychological support, mean (SD)	31.9 (0.5)	34.3 (0.2)	33.1 (1.7)	<.001
Physical independence, mean (SD)	22.2 (0.5)	23.2 (0.4)	22.7 (0.6)	<.001
Pain, mean (SD)	32.7 (0.6)	33.4 (0.5)	33.1 (0.4)	<.001

^a^DHM: digital home monitoring.

^b^QoR-40: 30-day quality of recovery.

Responses from the patient and caregiver satisfaction surveys administered at the end of the fourth week postoperatively were recorded (Multimedia Appendices 2 and 3, respectively). All 37 patients who completed the intervention answered the program satisfaction survey questionnaires (100%; 95% CI 90.5%-100%). More than 95% of patients agreed or strongly agreed that the instructions for setting up the remote monitoring system were easy to understand. All 37 patients in the DHM group agreed or strongly agreed that they felt safe at home and that the CN and physicians responded promptly and efficiently. All patients in the DHM group either agreed or strongly agreed that they would recommend the remote monitoring system program to future patients. Out of 80 caregivers who provided consent for enrollment, only 36 caregivers responded to the caregiver satisfaction questionnaires at the end of the fourth week post hospital discharge (47.7%; 95% CI 35.7%-59.1%). While taking care of the family members at home after the hospital discharge, our sample caregivers reported less burden on family members (8.5%), less interference with personal activities (28.5%), feeling less confined to staying at home (37.1%), and less physical strain (14.2%). However, caregivers reported taking more time off work than initially anticipated (14.2%), employment activities being affected (14.2%), educational activities being affected (8.5%), increased demand on time (31.4%), changes in personal plans (51.4%), and family adjustments (62.8%). Health care providers reported a 100% satisfaction rate (Multimedia Appendix 4).

## Discussion

The findings from this trial support the feasibility of conducting a full-scale RCT to compare DHM with the current standard of care after thoracic surgery. The study showed excellent feasibility, achieving a recruitment rate of 87.9%, protocol adherence of 96.3%, and collecting outcome data for 98.7% of participants. These results indicate significant engagement and compliance, reinforcing the study's viability for broader implementation.

The most common barrier to participation among eligible patients in this study was a lack of willingness to participate in research while receiving care (n=5, 5.4%). Other reasons included patients who felt they had enough support at home to recover after hospital discharge (n=4, 4.3%). However, all patients consented to randomization due to the preconception that the care team would connect with them after hospital discharge to aid their recovery. This finding suggests that recruitment for a full-scale trial may be facilitated by addressing implicit biases and emphasizing the importance of continuous connection with the care team to improve postoperative outcomes. Most patients preferred being assigned to the continuity of care with a DHM group rather than the standard care group (70%). In comparison, 25% of the patients did not express any preference.

Using smartphone technology for postoperative follow-up and patient communication has significantly minimized the chances of ED visits and RRs [33,34]. In the United Kingdom, a remote monitoring initiative for 900 colorectal patients reduced costs by 63% while achieving high patient satisfaction [35]. Likewise, a quality improvement study involving 48 thoracic patients with robotic lobectomies found that home monitoring effectively enabled safe early discharges and demonstrated possible economic benefits [36]. Conversely, in an RCT that included 292 postsurgical patients, there was no notable difference between the home monitoring and control groups in ED visits post surgery. Patients in the remote patient monitoring group had an average adherence rate of 86% for daily vital sign logging and 78% for daily question logging [37]. Still, home monitoring was well-received by both patients and physicians, although technological challenges diminished its benefits. Many of these studies relied on automatic data collection methods. Our research yields similar findings but is a prospective RCT focused on thoracic surgical patients. We incorporated more pragmatic inclusion criteria with the caregivers' surveys and used Vivify technology. Our intervention is labor-intensive, differing from other studies, including educational resources, automated questionnaires, vital sign data collection, 2-way communication, and daily CN calls.

This pilot RCT examined the feasibility and clinical impact of continuous DHM on postoperative outcomes in patients undergoing major thoracic surgery. The DHM group had fewer postoperative complications, unplanned ED visits, and unplanned RRs. A potential explanation may be the increased continuity of care and the clinician's ability to monitor a patient's clinical status to implement necessary interventions before the progression of postoperative complications or ED visits [38-41]. Moreover, the global QoR-40 score and all individual domains were rated higher in the DHM group. This may have resulted from increased patient surveillance and clinician intervention to ensure patients remain on an acceptable path to recovery [42]. However, this trial was not powered or designed with the QoR-40 scores as a primary outcome; thus, these findings must be interpreted cautiously. The satisfaction survey results indicate that patients and health care providers highly value the remote monitoring program. However, caregivers have shown mixed responses. Our findings imply that while patients and providers regard the program positively, further support for caregivers could improve their experience and address the reported increased time demands and schedule adjustments. This can be explored further in the full-scale RCT.

A potential barrier to implementing a DHM system is the difficulty of setting it up and using it by the patient. However, in our study, most patients reported that setup instructions were easy to understand and did not find the system difficult to use. Overall, satisfaction with the program was excellent, and all participants would recommend the remote monitoring system to future patients. Of note, caregivers of patients in the DHM group reported that caregiving affected their personal, educational, and work activities more than the control group. This may be explained by the need to assist the patient in recording vitals and concerns and uploading this information to the digital care platform.

One strength of our study was the diverse patient population regarding gender, age, and BMI. Pathologies such as primary malignancies, secondary malignancies, and nonmalignant diseases were also included. Surgical procedures were diverse, with various types of resections and surgical approaches. The heterogeneity of the study patients indicates that this can be universally implemented in other surgical populations. Patients and their caregivers were adequately trained to record vital signs and upload concerns online, reducing the workload of the home care team. Furthermore, extensive remote patient monitoring was implemented, including HR, NIBP, SpO_2_, and daily assessment measurements.

The limitations of this study include the fact that it was not statistically powered to detect postoperative outcome differences. As such, any between-group comparison should be interpreted with caution. Additionally, patients were only followed for 4 weeks postoperatively, so data on the efficacy of continuous DHM on postoperative outcomes beyond this time point remain unknown. Since January 2023, Vivify technology has not been available in Ontario, Canada, and we will be using different technology in our next project to explore these promising results. The potential threats to this feasibility may be reproducibility and scalability associated with the entirely new platform and the maintenance of labor-intensive resource intervention. Further, the cost of the intervention should have been evaluated in this study. Finally, this study was performed at a single center in patients undergoing major thoracic surgery and may need exploration to implement in other surgical populations at different institutions.

In conclusion, the VivifyHealth digital health platform provides a user-friendly interface to extend continuity of care. DHM effectively improved the quality of patients' recovery while decreasing postoperative complications, unplanned ED visits, and hospital RRs. Effective implementation of these platforms may reduce the utilization of scarce health care resources while maintaining excellent patient outcomes and satisfaction. Findings from this pilot trial support the feasibility of conducting a robust full-scale trial to explore these promising results.

## Appendix

Multimedia Appendix 1 Health problems addressed via a digital home monitoring program.

Multimedia Appendix 2 Patient/Program Satisfaction Questionnaires.

Multimedia Appendix 3 Caregivers Satisfaction.

Multimedia Appendix 4 Healthcare Provider satisfaction.

Multimedia Appendix 5 CONSORT eHEALTH checklist (V 1.6.1).

## Abbreviations

CN: clinical navigator
CONSORT: Consolidated Standards of Reporting Trials
CONSORT-EHEALTH: Consolidated Standards of Reporting Trials of Electronic and Mobile Health Applications and Online Telehealth
CTAS: Canadian Emergency Department Triage and Acuity Scale
DHM: digital home monitoring
ED: emergency department
HR: heart rate
NIBP: noninvasive blood pressure
QoR-40: 30-day quality of recovery
RCT: randomized controlled trial
REDCap: Research Electronic Data Capture
RR: readmission rate
SpO2: hemoglobin oxygen saturation
