# Supplementary figures and images for: Enhancing Quadruple Health Outcomes After Thoracic Surgery: Feasibility Pilot Randomized Controlled Trial Using Digital Home Monitoring
Source: JMIR Perioper Med. 2025 Feb 12;8:e58998. doi: 10.2196/58998 (PMC11888079; doi:10.2196/58998)

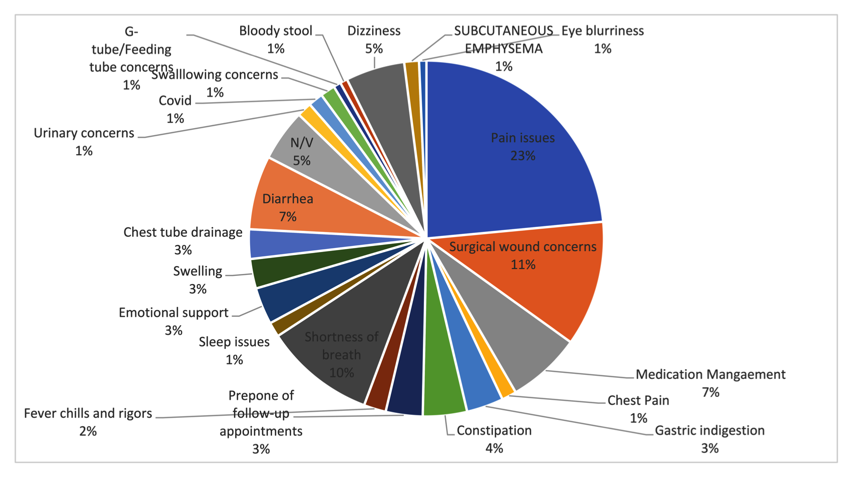

Supplement: Multimedia Appendix 1 [file periop_v8i1e58998_app1.png]
