# Supplementary material for: Enhancing Quadruple Health Outcomes After Thoracic Surgery: Feasibility Pilot Randomized Controlled Trial Using Digital Home Monitoring
Source: JMIR Perioper Med. 2025 Feb 12;8:e58998. doi: 10.2196/58998 (PMC11888079; doi:10.2196/58998)
Supplement: Multimedia Appendix 2 [file periop_v8i1e58998_app2.docx]

**Enhancing Quadruple Health Outcomes after Thoracic Surgery: A Feasibility Pilot Randomized Controlled Trial Utilizing Digital Home Monitoring.**

**Appendix 1 – Patient/Program Satisfaction Survey**

**STRONGLY DISAGREE=1 to STRONGLY AGREE=5 using a checkmark (✓) or an X**

|  | **Program satisfaction Questionnaire (n=37)** | **STRONGLY DISAGREE**  **1** | **DISAGREE**  **2** | **NEITHER AGREE OR DISAGREE**  **3** | **AGREE**  **4** | **STRONGLY AGREE**  **5** |
| --- | --- | --- | --- | --- | --- | --- |
| Q1 | "The information provided told me what to expect about the Remote Monitoring System at home." | 0% | 2.7% (1) | 0% | 8.1% (3) | 89.1% (33) |
| Q2 | "The instructions on how to set up and use the Remote Monitoring System were easy to understand." | 0% | 5.4% (2) | 0% | 16.2% (6) | 78.3% (29) |
| Q3 | "The Remote Monitoring System was difficult to use." | 75.6% (28) | 13.5% (5) | 2.7% (1) | 5.4% (2) | 2.7% (1) |
| Q4 | "I felt safe at home during the 30 days of monitoring" | 0% | 0% | 0% | 5.5% (2) | 94.4% (34) |
| Q5 | "During the 30-day monitoring, the response by the Clinician was efficient." | 0% | 0% | 0% | 2.7% (1) | 97.2% (36) |
| Q6 | "There was too much to manage at home including the Remote Monitoring System." | 78.3% (29) | 13.5% (5) | 2.7% (1) | 2.7% (1) | 2.7% (1) |
| Q7 | "The length of 30-days for the actual monitoring was just right" | 2.7% (1) | 2.7% (1) | 0% | 16.2% (6) | 78.3% (29) |
| Q8 | "During the 30-day monitoring, I would have liked more feedback from the Clinician " | 78.3% (29) | 5.4% (2) | 0% | 2.7% (1) | 13.5% (5) |
| Q9 | "I would recommend the Remote Monitoring System program to future patients" | 0% | 0% | 0% | 5.4% (2) | 94.5% (35) |
