# Supplementary material for: Enhancing Quadruple Health Outcomes After Thoracic Surgery: Feasibility Pilot Randomized Controlled Trial Using Digital Home Monitoring
Source: JMIR Perioper Med. 2025 Feb 12;8:e58998. doi: 10.2196/58998 (PMC11888079; doi:10.2196/58998)
Supplement: Multimedia Appendix 3 [file periop_v8i1e58998_app3.docx]

**Enhancing Quadruple Health Outcomes after Thoracic Surgery: A Feasibility Pilot Randomized Controlled Trial Utilizing Digital Home Monitoring.**

**Appendix 2 – Caregiver’s Satisfaction Survey**

|  | **Caregiver’s Questionnaire’s** | **Total**  **n=36 (%)** | **Control Group**  **n=16 (%)** | **DHM Group**  **n=20 (%)** |
| --- | --- | --- | --- | --- |
| 1 | My sleep was disturbed | 37.1 | 33.3 | 40 |
| 2 | It was manageable/reasonably comfortable at home after discharge from the hospital | 91.4 | 93.3 | 90 |
| 3 | It was a physical strain | 14.2 | 20 | 10 |
| 4 | I was confined to staying at home | 37.1 | 40 | 30 |
| 5 | There were family adjustments | 62.8 | 46.6 | 75 |
| 6 | There were changes in personal plans | 51.4 | 46.6 | 55 |
| 7 | There were other demands on my time | 31.4 | 26.6 | 35 |
| 8 | There were emotional adjustments | 74.2 | 80 | 70 |
| 9 | There were work adjustments | 17.1 | 20 | 15 |
| 10 | There was a financial strain | 14.2 | 6.6 | 20 |
| 11 | It was overwhelming for me to provide care in the home atmosphere | 11.4 | 13.3 | 10 |
| 12 | Leisure or recreational activities were affected | 37.1 | 46.6 | 30 |
| 13 | Educational activities were affected | 8.5 | 6.6 | 10 |
| 14 | Employment activities were affected | 14.2 | 13.3 | 15 |
| 15 | Extra time off work was taken than originally anticipated | 14.2 | 10 | 20 |
| 16 | Taking care of the patient interfered with my activities | 28.5 | 40 | 20 |
| 17 | Taking care of the patient placed the burden on our own immediate family members | 8.5 | 13.3 | 5 |
