# Supplementary material for: Enhancing Quadruple Health Outcomes After Thoracic Surgery: Feasibility Pilot Randomized Controlled Trial Using Digital Home Monitoring
Source: JMIR Perioper Med. 2025 Feb 12;8:e58998. doi: 10.2196/58998 (PMC11888079; doi:10.2196/58998)
Supplement: Multimedia Appendix 4 [file periop_v8i1e58998_app4.docx]

**Enhancing Quadruple Health Outcomes after Thoracic Surgery: A Feasibility Pilot Randomized Controlled Trial Utilizing Digital Home Monitoring.**

**Appendix 3 – Healthcare Provider Satisfaction**

**STRONGLY DISAGREE=1 to STRONGLY AGREE=5 using a checkmark (✓) or an X**

|  | **Healthcare Satisfaction Questionnaire (n=9)** | **STRONGLY AGREE** |
| --- | --- | --- |
| Q1 | What is your role in the Remote Care Monitoring Program? | Monitoring Team |
| Q2 | The technology I used to monitor patients was easy to use | 100% |
| Q3 | The features in the technology enable me to perform my work efficiently | 100% |
| Q4 | The remote monitoring program enables me to meet my patient's needs | 100% |
| Q5 | The technology enhances our ability to coordinate the continuity of care | 100% |
| Q6 | The information in the technology allows me to make patient care decisions and/or recommendations more quickly | 100% |
| Q7 | The technology helps me follow changes in the patient’s clinical condition over time | 100% |
| Q8 | I feel confident in my skills to provide care virtually | 100% |
| Q9 | Overall, how satisfied were you with the remote monitoring program? By “satisfied”, we mean the ease and functionality of the solution, quality of information, and services provided. | 100% |
